# A Tactile Skin System for Touch Sensing with Ultrasound Tomography

**DOI:** 10.3390/s23136071

**Published:** 2023-07-01

**Authors:** Manuchehr Soleimani, Tomasz Rymarczyk

**Affiliations:** 1 Engineering Tomography Laboratory, Department of Electronic and Electrical Engineering, University of Bath, Claverton Down, Bath BA2 7AY, UK; 2 Research & Development Centre Netrix S.A., Poland & WSEI University, Wojciechowska 31, 20-704 Lublin, Poland; tomasz@rymarczyk.com

**Keywords:** ultrasound tomography, soft skin, artificial skin, touch sensing

## Abstract

The tomographic imaging method is promising in large-area touch-sensing applications. This paper presents a new type of such touch sensor using ultrasonic tomography (UST) via sound attenuation imaging. UST is gaining popularity as a portable, fast, and inexpensive imaging system for medical and industrial applications. UST can be developed in different operation modes. A transmission mode UST is being investigated as a force- and touch-sensitive skin. A prototype skin sensor was developed in a 200 mm diameter circular UST array containing two sets of 16 transducers, with one operating at a central frequency of 40 kHz and the other at 300 kHz. The extension of the sensor in terms of dimension, up to 400 mm diameter, and number of sensors, up to 32 transducers, is possible where eight points of contact were reconstructed successfully. The medium contains a 20 mm high water region, and a soft silicone membrane covers the liquid region. When touchpoints or forces are applied to the soft skin of the membrane, the sound pathway is disrupted, resulting in an image of the touch position and touch force intensity using a tomographic UST algorithm. Several static and dynamic experiments are conducted to demonstrate this novel application of UST. In addition, a correlation analysis is carried out to establish the force quantification potential for the UST-based tactile skin.

## 1. Introduction

Ultrasound tomography has gained popularity in many new applications, mainly in medical imaging and industrial process monitoring. This work introduces a novel application of ultrasound tomography as a soft robotics touch sensor. New robotics applications such as manipulation, human–robot interaction, and healthcare [1,2,3] rely on a robust sense of touch. In these applications, detecting the force produced by touch is essential, which demands the reliable gathering of tactile information. There is a growing body of research into other industrial applications, such as structural health monitoring by real-time mapping of pressure and stress applied to structures [4] using skin-like sensors. A new touch and force sensor is proposed in this paper, based on ultrasound tomography (UST), with a liquid medium and a soft skin membrane. In addition, tomographic imaging methods, such as electrical impedance tomography (EIT), electrical capacitance tomography, and waveguide-based optical sensing, have shown promise in areas of artificial skin and touch sensing [5,6,7,8].

A tomographic-based reconstruction algorithm allows for efficiency on a data point and provides temporal and spatial information on touch sensing.

EIT, widely studied in this area, works by imaging the electrical conductivity mapping and measuring the trans-impedance on boundary electrodes. The sensors are fabric, carbon composites, or other electrical materials. While EIT is a good candidate for such an application, it suffers several challenges. The electromechanical properties in EIT-based sensing skins often challenge modelling and measurement. In addition, it leads to less reliable force and pressure evaluations, due to nonlinearity and hysteresis. Generally, extending EIT sensors to extensive areas is not feasible, as we need to either provide more excitation current or additional electrodes in central areas of the sensor.

Furthermore, the sensing resolution is low in the central area of a large EIT sensor, due to the challenges of a highly ill-posed inverse problem for image reconstruction. Recently, a liquid-based EIT skin was introduced [9] to overcome some of the electromechanical coupling issues in fabric-based EIT sensors, but low sensitivity in the central area, away from the electrode array, is still a challenge. Moreover, due to the soft field nature of EIT imaging, the image resolution cannot be improved by adding more sensors.

The closest tomographic-based skin to what is proposed here is the EIT-based skin. There are other emerging tomographic-based skins, such as optical and magnetic-based tomography, but there is not enough research data and literature to compare to. The EIT-based tactile skin is widely used due to its geometrical flexibility, which could be adapted to many applications. However, the EIT-based skin has several drawbacks [8]. For large areas of skin, the EIT will provide low-quality force and touch information in the central area of imaging. In the EIT, additional electrodes are also placed in central areas [8] to solve this problem. While this is helpful, in many applications, electrodes in the central area of the skin are not desirable. In addition, the number of electrodes limits the EIT resolution and, due to the ill-posed nature of EIT reconstruction, increasing the number of electrodes is not feasible due to signal quality.

This work shows a UST-based skin sensor, which can overcome some EIT-based skin limitations. UST has been used in many medical and industrial applications [10,11,12,13] and has been a successful imaging modality in medical applications. For example, in [13,14], UST was shown to work for breast cancer imaging, acting as a new modality which could work well, especially for denser breasts in replacement, or in addition to X-ray mammograms. UST has also been used in structural nondestructive testing [15] and industrial process monitoring [16,17]. UST shows great versatility and reliability in these applications, leading to a clinically approved or commercially viable industrial device. An essential property of UST is a very good resolution when many sensors are used, and the transmission-mode UST shows an exemplary imaging performance inside the object (or process). It makes the UST a good candidate for a large-area robotic skin, a novel method that will be demonstrated in this work.

The operation of UST is based on the analysis of the propagation of acoustic waves in different materials based on the distributions of sound velocity or pulse amplitude. It is intended to represent the acoustic properties of the medium. The transmission-mode UST is based on imaging properties, such as acoustic attenuation (AA) or time of flight (TOF), which can be used to determine the amplitude and velocity profiles of sound in the region of interest (ROI) through quantitative information. Image fusion uses the results of TOF-UST and AA-UST measurements [10]. Regarding the size of a UST-based skin, it can be implemented in a large area with a high sensitivity in the central area. Due to the less ill-posed nature of the inverse problem in transmission UST (for example, when compared to EIT), the image resolution can be improved by incorporating more sensors.

The article is arranged in the following way. Section 2 describes the UST detector and the reconstruction process, and provides reflection and transmission tomography of image reconstruction formulas. On the other hand, Section 3 presents and evaluates the experimental results, which include fixed single and multiple touch points, dynamic touch, and the quantification of a force of touch through UST imaging, as well as scaling up for both the size of the skin and the number of transducers. Finally, Section 4 provides some context on the results, focusing on the UST sensor’s scalability and the sensing medium’s application, and Section 5 contains the concluding remarks.

## 2. UST Imaging System

The UST measurement system used in this study includes the transmission-based time of flight (TOF) data and the amplitude attenuation (AA), allowing for two types of transmission measurement. In a 16-transducer system, every transducer acts as a transmitter in turn, and the remaining transducers are used for measuring the arrival time and amplitude of arrival signals. The sensors within a 120 degree field of view of the transmitter provide more reliable transmission data. The UST system can produce five frames of data per second. Such a frame rate will allow us to investigate the dynamic imaging behaviour. Two or three frames per second may be sufficient in many touch and force sensing applications. A faster frame rate is always useful, as it will allow an analysis of touch stimuli and their corresponding responses in skin sensors. In the following subsections, the sensor design and the UST image reconstruction are briefly described in the context of the application in this paper.

### 2.1. UST Sensor System

Measurements were made on a tank with a diameter of 200 mm and a height of 20 mm. The tank was filled with water, and a silicone membrane was glued to the top of the sensor. On the tank’s perimeter, 16 ultrasonic transducers at 40 kHz frequency with a diameter of 14 mm, and 16 transducers at 300 kHz with a diameter of 13 mm, are mounted. The centre of the transducers was mounted 9 mm from the bottom of the tank alternately, one behind the other, using hot glue. The height of the water-filled part of the tank from the bottom to the silicon membrane is 18 mm.

**Figure 1:**
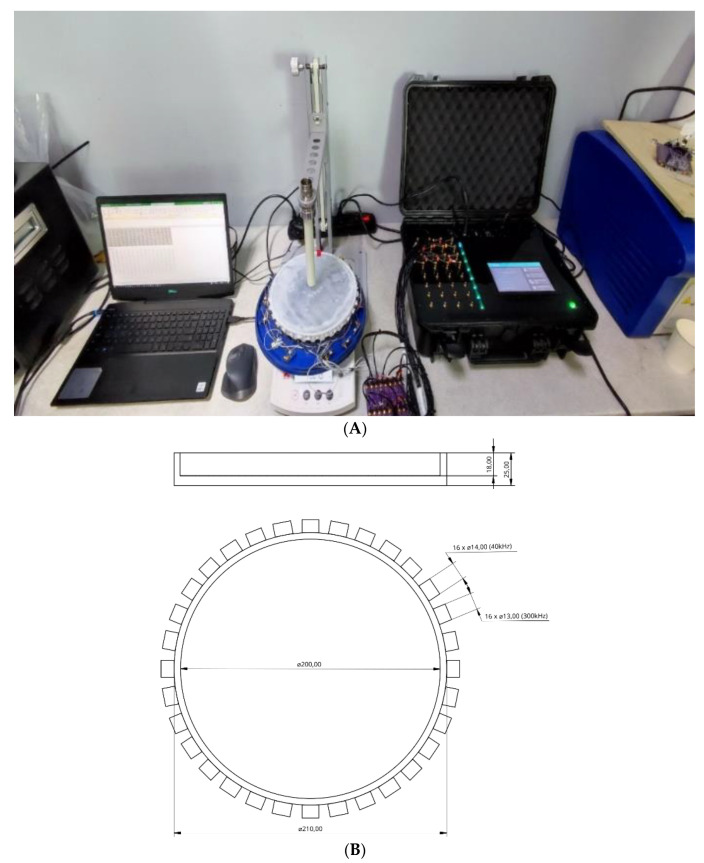
The experimental setup including (**A**) UST system and sensor and (**B**) skin sensor with dimensions.

**Figure 2:**
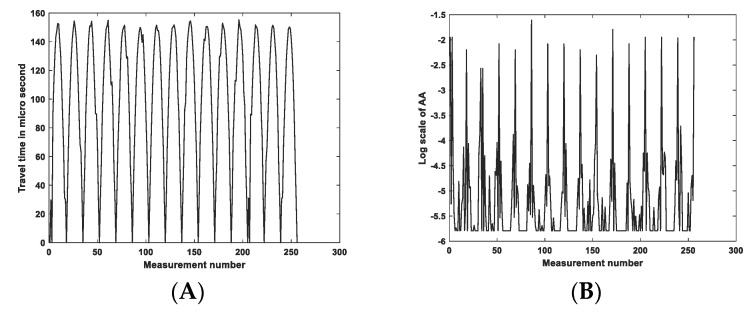
Background data for TOF and amplitude attenuation for 300 kHz excitation, (**A**) TOF background data, (**B**) AA background data.

The measurements taken were TOF and AA. Measurements made with 40 kHz converters may differ in value from 300 kHz converters due to the extended rise time of the signal (T = 25 µs for 40 kHz), so the conversion to the envelope has a larger rise time. For both frequencies, the frame rate of the UST device is five frames/s. However, the background and the measurement differences should be relatively similar for both types of transducers. Figure 1A depicts the UST system with data acquisition hardware, a data visualization computer, and the transducer array with the skin sensor. Figure 1B shows the sensor dimension accommodation of 16 sensors in each frequency. The reference data set when the sensor skin is in relaxed mode can be seen in Figure 2A,B, showing measurement sets of time of flight and amplitude attenuation. For 16 channel sensors, 256 measurements are possible, though the received signal from the transmitting transducer is discarded and set to 0 in the measurement data set. The AA is based on the amplitude or the arrival pulse, and the TOF is the arrival time of the first receiving signal in the receiving transducer. The AA value represents the amplitude loss when the signal travels through the sensor’s liquid domain without deformation in silicon-based skin. The mean signal-to-noise ratio (SNR) value for TOF data for 300 kHz is 61 dB, and 64 dB for 40 kHz, and the SNR for AA is 48 dB for 300 kHz, and 47 dB for 40 kHz. Transducer parameters for the 300 kHz sensor are as follows: the centre frequency—300 ± 15 kHz, received sensitivity echo (V)—≥800 mV (driven signal: 200 Vp-p, 300 kHz, at 20 cm), capacitance (pF)—300 ± 25% at 1 kHz, the distance of detection—0.04 m to 1 m, and operating temperature range from −20 °C to +80 °C.

### 2.2. UST Image Reconstruction

Transmission-mode UST imaging works by determining the difference between a relaxed mode as reference data, TOF_relaxed_ and touch data when the domain is disturbed due to an interaction with the skin, TOF_touch_. The TOF measurement data, differs TOF_diff_, is derived by subtracting the full measurement data from the background data and defining the travel time delays (μs), as shown by Equation (1).

TOF_diff_ = TOF_touch_ − TOF_relaxed_ (1)

(2) $${\mathrm{AA}}_{\mathrm{diff}}=\frac{1}{f_{c}}\ln\left(\frac{{\mathrm{AA}}_{\mathrm{relaxed}}}{{\mathrm{AA}}_{\mathrm{touch}}}\right)\;$$

where ${\mathrm{AA}}_{\mathrm{relaxed}}$ is the signal’s amplitude at each receiver when there is only water in the field of view (FOV) as reference data, and ${\mathrm{AA}}_{\mathrm{touch}}$ is the amplitude of the complete data. Here, $f_{c}$ is the center frequency of the excitation pulse.

(3) ΔM= S ΔX

(4) $$\Delta X\approx \;S^{T}\;\Delta M$$

Advanced iterative reconstruction algorithms such as total variation (TV) regularization are used, which have more potential to solve the regularized inverse problem fixedly, as described in [18]. Simple image averaging is used as a means of data fusion to combine the two forms of transmission images [10]. The average normalized intensities of TOF and AA images are combined.

The TV algorithm will produce an image of 64 × 64 pixels. Let us consider XTOF and X_AA_ as the reconstruction TOF and AA images. First, we normalize each image against the peak value in each image, so that both TOF and AA images have a max value of 1. We then use the average value of TOF and AA for each imaging pixel for the fusion image. It is the same approach used in [10]. In [10], a triple modality UST system was developed that included the reflection mode UST, while the reflection mode UST can also work for the UST-based touch skin. In this paper, the focus is on the transmission-mode UST.

## 3. Imaging Results

This section uses the proposed UST system and method to describe the skin sensing results in static imaging for single and multiple touch sensing, dynamic sensing, and quantitative force imaging. Extensive experimental work was carried out using the prototype system, where we show a selection of those results. In static touch sensing, the aim is to identify the touch(es) and their position observed from AA, TOF, and fusion images. In dynamic testing, the aim is to observe the temporal responses when the silicon-based skin is disturbed by an external single and multiple-touch stimulation. Finally, in force quantification, the experiments are carried out with a different level of touching force applied with a rod to establish a correlation between the applied force and the UST imaging responses.

### 3.1. Image of Static Touchpoints

**Figure 3:**
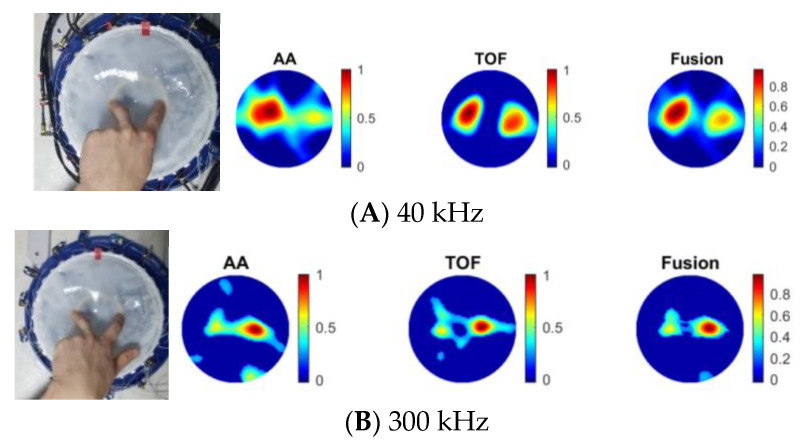
Sensing single touch point. (**A**) Single point, (**B**) two points.

**Figure 4:**
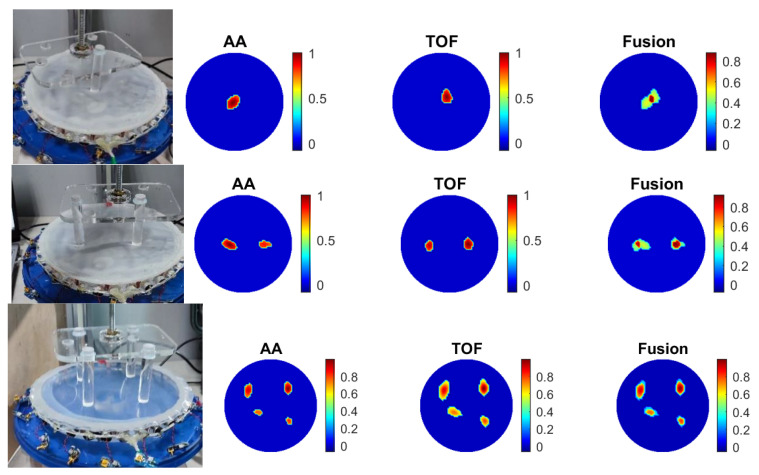
Sensing single and multiple touchpoints for 300 kHz excitation.

The experiments are performed with touch and pressure applied through the fingertip(s), and data are collected for both 40 kHz and 300 kHz sensor sets. Figure 3 shows the imaging results for such a fingertip touch sensing. A background data set is first collected, allowing reference data generation for AA and TOF imaging. In each case of touch force applied to the skin surface, 10 frames of data were collected. The average values of these 10 frames were used as the measurement data in each case. As shown in Figure 3, those touches were detectable in AA, TOF, and fusion images for both sets of frequencies. Figure 4 shows more examples of multi-touch sensing when 300 kHz frequency is used.

### 3.2. Image of Dynamic Touchpoints

**Figure 5:**
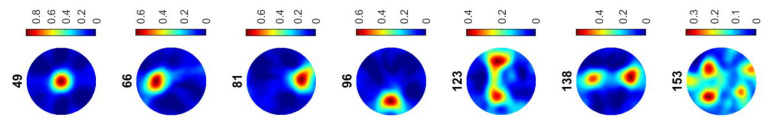
Dynamic images from single and multiple touch fusion images from selected frames.

**Figure 6:**
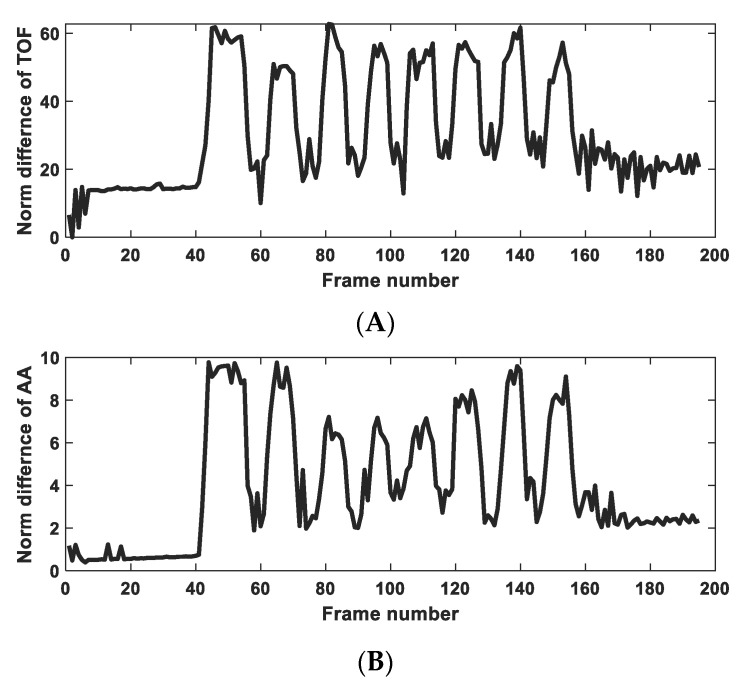
Tracking the measurement data via norm value for differences for all data collected in 196 frames. (**A**) TOF data, (**B**) AA data.

The real-time and dynamic response of the touch sensors is an important consideration to resemble natural touch and force sensing. Hence, several dynamic experiments were carried out to investigate the response of the skin sensor. In the case of dynamic testing, the first few frames are relaxed (reference data), and the remaining frames are analysed to track the dynamic sense of touch. One of these experiments is shown in Figure 5, where several single- and multiple-finger touches are applied to the skin membrane after early reference data sets with a 300 kHz sensor array. Sample images in Figure 5 show single points of touch, two points up and down, two points left and right, and contact with 4 points. Analysing the global change in all measurement data vs. the reference data shows the dynamic nature of the stimuli applied to the skin, as shown in Figure 6. The dynamic response of the sensing skin depends on both the frame rate of the UST device and the mechanical interaction between the membrane skin and the base liquid. This is because the sensor will respond to changes in the morphology of the surface of the skin due to the force applied to it. When the skin material (in this case) occupies the space occupied by the water, the changes in the speed of sound and the attenuation of the acoustic signals can be reconstructed as an image of touch and force sensing.

### 3.3. Quantitative Force Evaluation

**Figure 7:**
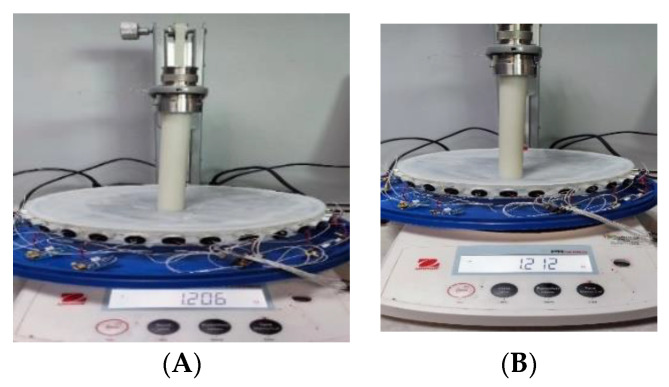
Force analysis set up with the soft UST sensor, an almost perpendicular force is recorded in Newtons, in this case (**A**) 1.206 N in the centre, and (**B**) 1.212 N in the corner.

Several experiments were carried out to quantify the AA and TOF imaging vs. the soft skin and force deformation. The pressure force was measured using an OHAUS PRSERIES scale, graduated in N. The force was step changed by ~0.2 N. We take full characteristics from 0–4 N in the arm’s position. A force of 4 N is needed to push the silicone membrane to the bottom of the tank. Figure 7 shows the experimental setup for force evaluation. Figure 7A shows a photograph of a force of 1.206 N applied to the central area of the sensor, and Figure 7B shows a force of 1.212 N applied to a corner area near the transducer number 9 for 300 kHz. While pressure applied to the central area creates a more homogenous deformation, the force applied to the corner area might be affected by the slightly different resistive force, due to the more rigid nature of the boundaries. Such a function will be seen in the following UST imaging results.

**Figure 8:**
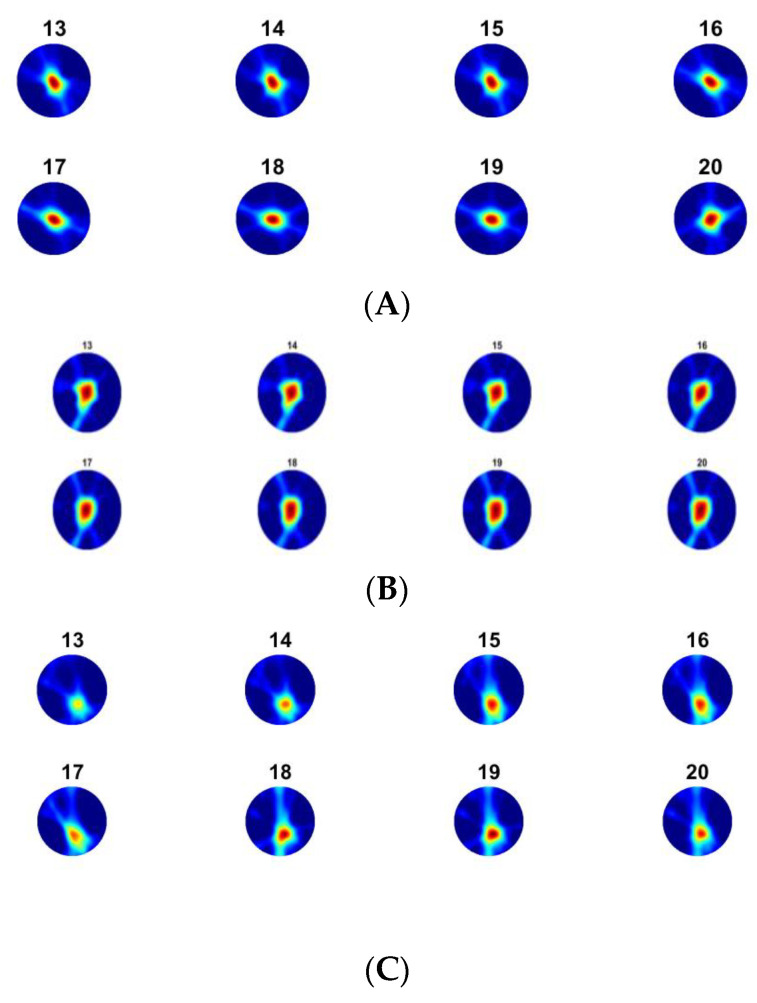

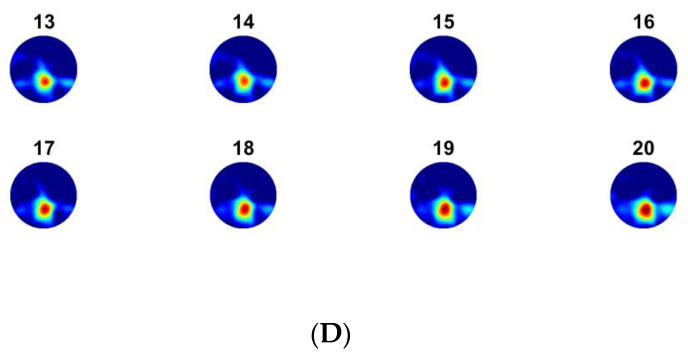
TOF image for 20 frames per increasing force, (**A**) TOF centre, (**B**) AA centre (**C**) TOF corner, (**D**) AA corner.

**Figure 9:**
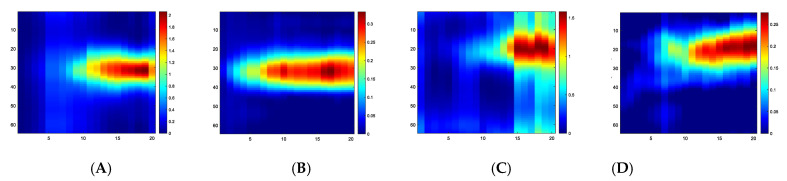
An image slice through 20 force points (*X*-axis) from TOF and AA images, (**A**) TOF centre, (**B**) AA centre, (**C**) TOF corner, (**D**) AA corner. Colour bar shows the relative scale in the image.

**Figure 10:**
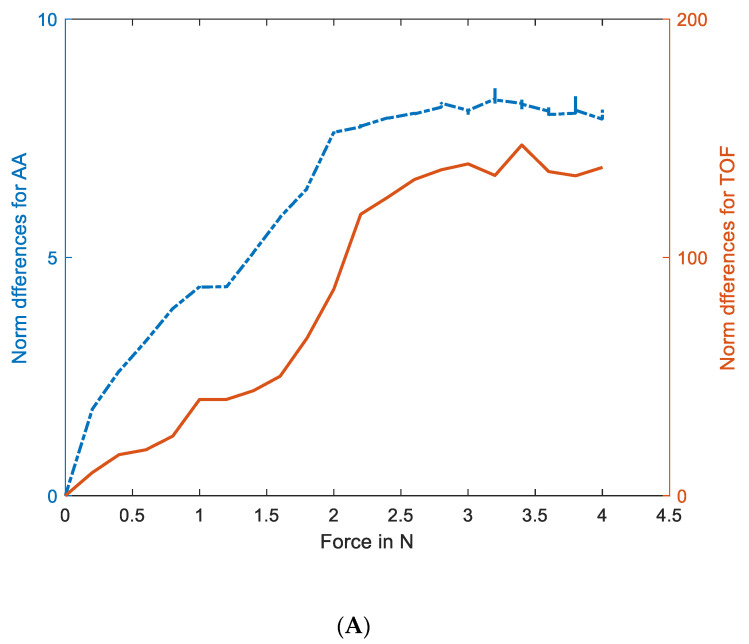

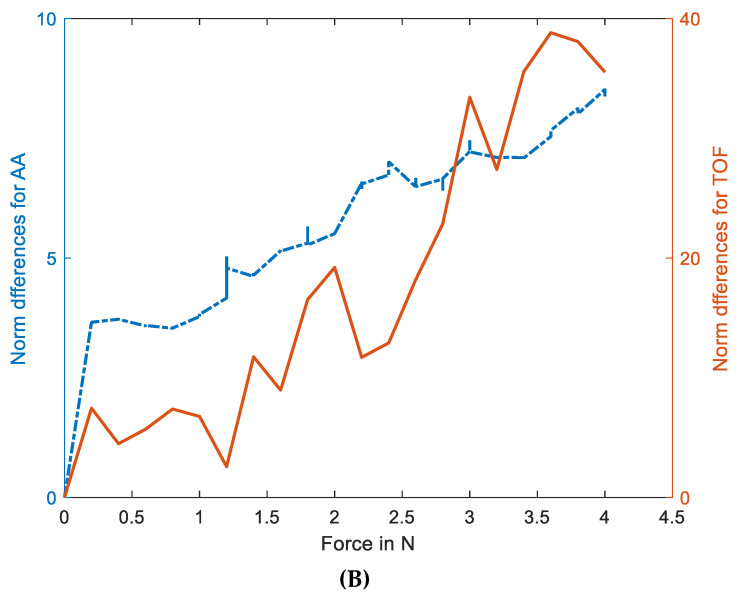
Norm differences for AA and TOF data show the data changes with forces, (**A**) centre, (**B**) corner. The result shows the relationship between force and UST measurement. Red line for ToF and blue line for AA.

Figure 8 shows the touch image’s position for 20 steps of force applied (we show only frames 13–20 here) to the central and corner areas. Considering the 20 force steps, Figure 9 shows the slice through 20 images. It shows a gradual increase of TOF and AA values in reconstructed images with increasing forces. In these experiments, a force as low as 0.2 N can be detected reliably by showing detectable variations in measured data and reconstructed images. However, at the other end of the force applied, when the rod approaches the bottom of the sensor pad, it is at the maximum level of disturbances in the UST area and, beyond that point, the extra force may not translate to more deformation on the skin. Figure 10 depicts the link between the actual force value and the UST data. A more robust correlation between the UST sensor and force can be seen in the central area of imaging. This correlation could generate calibration plots for future studies where pressure maps can also be produced. If the domain is of a compressive liquid and not water, further complex elastography studies would be needed to establish such a correlation.

**Figure 11:**
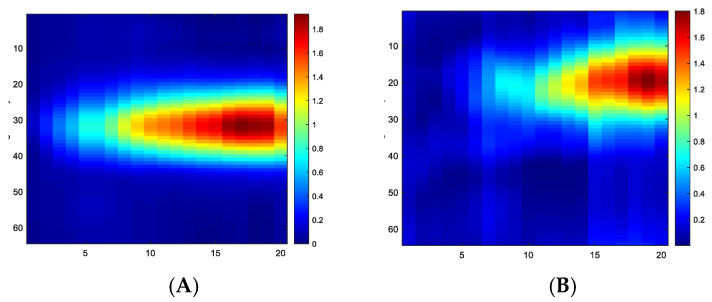
The force profile indicator with the fusion of TOF and AA images, (**A**) centre, (**B**) corner.

UST data shows that the central area touch sensing and force quantification are more robust. However, a force applied to the corner area, such as the one in Figure 9C, may produce a less robust image than the central area, as expected from a transmission-mode UST. Additionally, the differences in elastic forces, depending on the touch in the central area or corner, could result in different responses from UST sensor data, as the deformation may vary. Nevertheless, in terms of imaging sensitivity, this is the opposite of EIT-based skin sensing, suggesting a possibility of complimentary aspects between EIT and UST, if used together in multi-modality skin. Therefore, there are still some benefits in multi-modality AA and TOF imaging from the imaging point of view. Combining the profile of AA and TOF images with the same data fusion strategy, Figure 11 shows the fusion of the force profiles shown in Figure 9, depicting the force increase in a similar fashion to AA and TOF profiles. Although the quantitative analysis was carried out using AA and TOF images, the fusion image should provide a more reliable quantitative vs. force result.

### 3.4. Scaling the UST-Based Sensor

**Figure 12:**
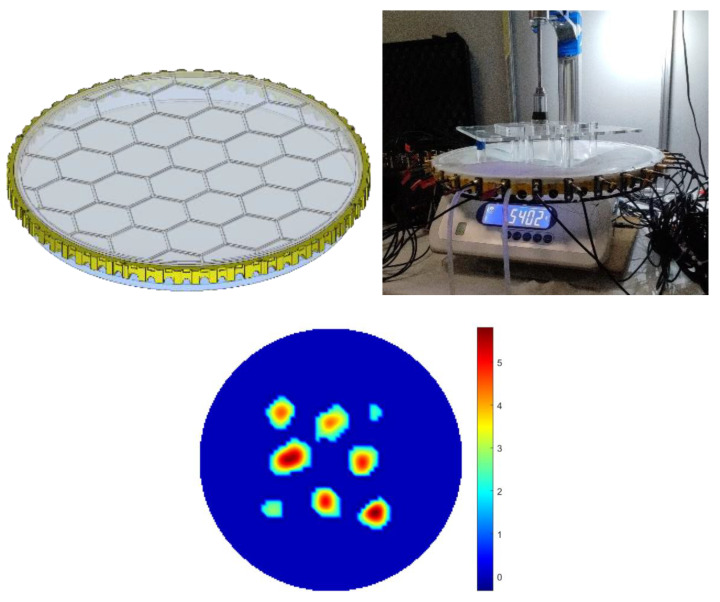
Reconstruction of eight force points with 32 channel sensor data with modified large area soft skin.

A skin area of 400 mm in diameter, with 32 sensors operating at 300 kHz, was developed to demonstrate the scalability of the UST-based skin in terms of the physical dimensions and the number of sensors. The sensors have a diameter of 13 mm and an overall height of 30 mm. In this case, the frame rate for the UST device is two frames/s. For large-area sensors, the membrane was made with 3D printing, creating a hexagon-type area where the edges of the hexagon are made of less elastic materials. As a result, it allows for more localized deformation and, consequently, higher-resolution touch sensing. Figure 12 shows a reconstruction of eight points of touch with the modified skin and 32 channel sensors.

In this experiment, for comparison purposes, we also used a 32-electrode EIT sensor where, as expected, the EIT sensors could not detect the eight force points in the central area.

## 4. Discussions

The UST-based sensor overcomes many challenges that exist in large-area skin development. Some areas in the UST could be used together with another imaging modality, such as EIT, as part of multimodality sensing. Referring to Figure 9 and Figure 10, the UST performs better in the central area of imaging, while both qualities of images and quantitative relations are more compromised near the boundary. This is the opposite of how an EIT-based sensor responds.

With more UST sensors, the image resolution can be further enhanced if much higher-resolution tactile sensing is needed. This is already the case in the medical imaging application of the UST [13]. However, the complexity of such a system will need to be considered and simplified to many-transducer systems re-designed for future skin applications. As shown in Figure 12, there is further opportunity to enhance the design surface skin interface to have the desired deformation responses.

The geometrical versatility of the UST-based sensor was not considered in this study. All experiments were on sensors sitting horizontally, making them a floor-based skin. In future studies, one could investigate a different kind of sensing medium, such as a gel-type material against water, and how the sensor could be used in cases such as wrapping around a robot’s arm. As the paper introduces a very exciting new tactile skin, many new studies could follow, both in terms of the basic principle of sensor operation and design, as well as its deployment and applications.

In the early prototype, 16 transducers for each frequency are used, a 32-channel system can significantly enhance the imaging resolution, and the sensor array can be made larger than the prototype shown here, allowing for more extensive area imaging. However, there is room for further improvement in the mechanical function of the interface membrane, especially if a large sensing area is needed. For this study, the pressure and temperature of the water medium do not change to a level that affects the signals [19,20], so the water medium performs well. Furthermore, unlike a previous study [21], the presented solution works on tomographic principles, where measurements are taken on the outer edge of the object under study, and an inverse problem is solved, resulting in image reconstruction.

The main purpose of the research presented in this article was to develop a reconstruction and measurement system for data analysis using ultrasonic transmission tomography. It was performed by designing a tomographic device and proprietary algorithms capable of reconstructing 2D images regardless of the size, shape, location, or number of inclusions hidden in the examined object. The presented measurement method uses the information in the ultrasonic signal after passing through the tested object. Ultrasonic waves, because they belong to short waves, have propagation and radiation properties, thanks to which ultrasound can be treated as rays. Therefore, measurements are continuously made online, and are not as invasive as well-tested tomographic solutions, e.g., CT. During such measurements, we can analyse changes in deformations of the tested object online on an ongoing basis, through the pressure force interpreted as a larger or smaller inclusion, depending on its value. For this purpose, the authors prepared an appropriate measurement model with a proprietary tomograph, adapted to the membrane with sensors by optimizing the measurement parameters and developing the research methodology and algorithms to solve the reverse problem.

The study aimed to verify whether ultrasonic tomography would sufficiently detect deformations on the surface of the test object. Such deformations can be increased or decreased by exerting a specific pressure force (which can be measured). From an algorithmic point of view, by solving the inverse problem, we detect inclusions of a certain size. Therefore, the relationship between tomographic measurements of ultrasound (TOF, AA) and the applied force is relative and, to some extent, reflects the size of the inclusion.

Here, we summarise the novel contributions of this article.

(1) In terms of the size of the skin, the UST has enhanced performance in central imaging areas that are very hard to achieve with electrical tomography-based sensors, unless you put more sensors in the central area of the large-size sensor. There is a practical argument against putting EIT electrodes in the central imaging area as it would affect the working of the skin;
(2) It is well established in medical UST that an increasing number of UST transducers will enhance image resolution, something that is not possible with EIT-based sensors. We demonstrated that in our paper, with eight points of contacts being detected when we increased the number of sensors from 16 to 32;
(3) Using a shallow liquid-based sensor will offer a self-healing effect for skin sensors;
(4) A novel surface 3-D print is innovative and, while the mechanical modelling for such a surface needs to be invested in the future, it is very innovative;
(5) A large number of results, with various degrees of accuracy and success, shows our transparency in reporting our results.

## 5. Conclusions

The paper introduces ultrasound tomography as a novel method for sensing touch and pressure. A prototype skin sensor was developed to evaluate such an application experimentally. A soft and flexible silicon-based skin membrane was used to act as an interface skin. Multi-touch static and dynamic tests were carried out to demonstrate the performance of such a sensor using the operational transmission mode. The tests were repeated in both 40 kHz excitation frequency and 300 kHz operational frequencies, where the latter shows more robust data for sensing touch. We demonstrate both a time-of-flight delay (speed of sound imaging) and an amplitude attenuation imaging mode, as well as their combination in an image fusion mode. Both sets of UST for AA and TOF work well, and some enhancement can be seen when a simple image fusion is applied. A quantitative force evaluation was carried out. A disturbance at 0.2 N could already be detected by image reconstruction. Although the correlation was observable between the force and the image (and UST) data that was quantified, a degree of force saturation occurs when the maximum force exerted through the pressure rod reaches the bottom of the sensor system. The design of the membrane skin and the depth of liquid in the skin sensor are important factors. The membrane needs to be designed accordingly, to achieve the desired range of force response.

The main advantage of tomographic examinations is their non-invasive measurement, which does not cause changes in physical and chemical parameters. The measurement information processed in the reconstruction process results in the visualization of the analysed objects. A significant difficulty in using ultrasonic tomography is that the measurements are strongly disturbed by gas bubbles in the examined areas, and the increased attenuation of ultrasonic waves with their frequency. The information obtained in the measurement process is merged along the measuring path of the ultrasonic wave, which affects their mapping and visualization. As a result of the tests, it can be concluded that the reconstructions correctly reflect the locations of the inclusions.

Nevertheless, a good indication of the correlation between UST data and the increase in forces (as an inclusion) can be observed. Increasing the number of UST sensors from 16 transducers to 32 or more can enhance image resolution. However, it will need to be balanced against the complexity and cost of the measurement system. UST-based skin can be implemented in larger area sensing, and the liquid nature of the domain in the skin allows a rapid self-recovery, making it an exciting new direction for touch sensing. In addition, the self-recovery mechanism offered by a liquid-based sensor makes the UST system robust to component failure. These results open the path to a new generation of versatile distributed soft skins due to their inherent size, resolution, robustness, and scalability. A multimodality sensor skin with EIT imaging could be interesting, and will be the topic of our continued study. Future work will also involve the implementation of a modified version of the algorithms, and the use of machine learning and deep learning methods, especially in the aspect of hybrid connections. According to the authors, this will increase the results’ quality and precision.

## Data Availability

Data can be provided upon the request to the authors.
